# Increased hepatic oxidative metabolism distinguishes the action of Peroxisome proliferator-activated receptor δ from Peroxisome proliferator-activated receptor γ in the *ob*/*ob *mouse

**DOI:** 10.1186/gm115

**Published:** 2009-12-07

**Authors:** Lee D Roberts, David G Hassall, Deborah A Winegar, John N Haselden, Andrew W Nicholls, Julian L Griffin

**Affiliations:** 1 Department of Biochemistry, University of Cambridge, Tennis Court Road, Cambridge, CB2 1QW, UK; 2 GlaxoSmithKline, Investigative Preclinical Toxicology, Park Road, Ware, SG12 0DP, UK; 3 GlaxoSmithKline, 5 Moore Drive, Research Triangle Park, NC 277709-3398, USA

## Abstract

**Background:**

The peroxisome proliferator-activated receptors (PPARs) are ligand-activated transcription factors and members of the nuclear receptor superfamily. The PPAR family consists of three members: PPARα, PPARγ, and PPARδ. PPARδ controls the transcription of genes involved in multiple physiological pathways, including cellular differentiation, lipid metabolism and energy homeostasis. The receptor is expressed almost ubiquitously, with high expression in liver and skeletal muscle. Although the physiological ligands of PPARδ remain undefined, a number of high affinity synthetic ligands have been developed for the receptor as a therapeutic target for type 2 diabetes mellitus, dyslipidemia and the metabolic syndrome.

**Methods:**

In this study, the metabolic role of PPARδ activation has been investigated in liver, skeletal muscle, blood serum and white adipose tissue from *ob*/*ob *mice using a high affinity synthetic ligand and contrasted with PPARγ activation. To maximize the analytical coverage of the metabolome, ^1^H-nuclear magnetic resonance (^1^H-NMR) spectroscopy, gas chromatography-mass spectrometry (GC-MS) and ultra performance liquid chromatography-mass spectrometry (UPLC-MS) were used to examine metabolites from tissue extracts.

**Results:**

Analysis by multivariate statistics demonstrated that PPARδ activation profoundly affected glycolysis, gluconeogenesis, the TCA cycle and linoleic acid and α-linolenic acid essential fatty acid pathways.

**Conclusions:**

Although activation of both PPARδ and PPARγ lead to increased insulin sensitivity and glucose tolerance, PPARδ activation was functionally distinct from PPARγ activation, and was characterized by increased hepatic and peripheral fatty acid oxidative metabolism, demonstrating the distinctive catabolic role of this receptor compared with PPARγ.

## Background

The peroxisome proliferator-activated receptors (PPARs) are ligand-activated transcription factors that control the expression of genes involved in organogenesis, inflammation, cell differentiation, proliferation, and lipid and carbohydrate metabolism [1,2]. A number of synthetic compounds used to treat type 2 diabetes and dyslipidemia are PPAR ligands. Upon binding their ligands, PPARs heterodimerize with the 9-cis-retinoic acid receptor and then bind to target gene peroxisome proliferator response elements, a direct repeat of the sequence AGGTCA separated by one nucleotide [3].

Three distinct subtypes of PPARs have been identified, PPARα, PPARδ and PPARγ, each demonstrating its own specific tissue distribution and ligand preference [4]. PPARδ is expressed almost ubiquitously, though some tissues express higher concentrations of the mRNA, including the brain, adipose tissue, skin, liver and skeletal muscle [5,6]. In addition, PPARδ protein expression has recently been shown to be high in liver, colon, small intestine and keratinocytes [7]. The receptor is activated by several 14- to 18-carbon-containing polyunsaturated fatty acids, including eicosanoids such as prostaglandin A_1_, iloprost and carbaprostacyclin [8].

In comparison to PPARα and PPARγ, PPARδ has been the focus of far less research, despite its potential clinical role. This is in part because only relatively recently have high affinity synthetic PPARδ ligands been developed that may be used for the treatment of the metabolic syndrome. Insulin-resistant obese rhesus monkeys treated with the selective PPARδ agonist GW501516 demonstrated significant increases in high-density lipoprotein (HDL) cholesterol with concomitant decreases in triglycerides and low-density lipoprotein cholesterol [9]. PPARδ activation reduced adiposity by decreasing intracellular triglyceride accumulation in mouse brown adipose tissue and liver and also enhanced β-oxidation in 3T3-L1 mouse preadipocytes [10]. PPARδ mRNA is expressed at 10 and 50 times the concentrations of PPARα and PPARγ mRNA [11], respectively, in skeletal muscle and administration of PPARδ agonists to rodents results in an increase in expression of genes involved in fatty acid oxidation, mitochondrial respiration, oxidative metabolism and slow twitch contractile apparatus, decreasing muscle fatigability [12]. However, the ubiquitous expression of PPARδ may result in diverse and unwanted side effects upon activation of this receptor. The nuclear receptor has been implicated in the acceleration of intestinal adenoma growth and increased growth in breast and prostate cancer cell lines, but conversely it also attenuates colon cancer [13-15]. The role of PPARδ in development and carcinogenesis is complex and has been previously reviewed [16]. PPARδ activation has also been implicated as a cause of muscle atrophy [17].

It is hypothesized that the insulin sensitizing effects of PPARδ activation are brought about by changes in systemic metabolism. Given that triglyceride in liver contributes to insulin resistance, it has been suggested that increased triglyceride oxidation in the liver, caused by PPARδ activation, may contribute to this improvement [18]. Therefore, this study aims to use metabolomics to examine the changes that occur in hepatic metabolism following PPARδ activation and the impact detected systemically through metabolite changes in blood serum and skeletal muscle in the *ob*/*ob *mouse. The *ob*/*ob *mouse model of insulin resistance is robust, well characterized and used extensively to study type 2 diabetes and its therapies; however, it is worthy of note that it is a monogenic paradigm of leptin deletion, whereas type 2 diabetes mellitus is a polygenic disorder. This study has used metabolomics in conjunction with traditional clinical chemistry end points to investigate the effects of a PPARδ agonist in contrast to a PPARγ agonist on liver, skeletal muscle, serum and white adipose tissue in the *ob*/*ob *mouse.

## Methods

### Clinical chemistry

All clinical chemistry measurements were performed using an Olympus AU 400e Analyzer [19]. Insulin measurements were performed by ELISA (Millipore Mouse Insulin ELISA kit, Billerica, MA, USA).

### Oral glucose tolerance test

Animals were fasted overnight prior to the oral glucose tolerance test (day 12). Glucose concentrations were measured using the FreeStyle Blood Glucose Monitoring System (TheraSense, Fleet, UK). Animals were dosed orally with 1 g/kg glucose. Baseline fasted glucose values were collected at the 0 minute time point. Glucose concentrations were collected at 15, 30, 60 and 90 minute intervals. All time points were collected via tail snips.

### Tissue collection and extraction

All animal studies were performed within the relevant local legislation. Two-month-old male *ob*/*ob *mice (Jackson Labs, Bar Harbor, ME, USA), with no significant variation in initial body weight (data not shown), were fed standard laboratory chow *ad libitum *under controlled temperature and lighting (20-22°C, 12-h light-dark cycle). The *ob*/*ob *mice were assigned to three groups of eight and dosed orally daily at 8 am with 0.5% hydroxypropylmethylcellulose/0.1% Tween80 vehicle control, a PPARδ agonist, GW610742 (30 mg/kg), and a PPARγ agonist, GW347845 (5 mg/kg). Injection volume was adjusted daily according to body weight at 10 ml/kg. Serum was collected via cardiac stick under isoflourane anesthesia at completion of the study (day 15). Skeletal muscle (gastrocnemius), liver and white adipose tissue were rapidly dissected (<60 s post mortem), snap frozen in liquid nitrogen and stored at -80°C until extraction.

Metabolites were extracted from tissues using a modified Bligh and Dyer method [20]. Frozen tissue (approximately 100 mg for nuclear magnetic resonance (NMR) and approximately 50 mg for gas chromatography-mass spectrometry (GC-MS) analysis) was pulverized with liquid nitrogen. Methanol-chloroform (600 μl; 2:1 v/v) was added to the pulverized tissue or serum (50 μl) and the samples were sonicated for 15 minutes. Chloroform-water (1:1) was then added (200 μl of each). Samples were centrifuged (16,100 g, 20 minutes) and the two phases were separated; the organic phase was dried in a fume hood; the aqueous phase was dried in an evacuated centrifuge.

### ^1^H-NMR spectroscopy

Dried extracts were dissolved in 600 μl of D_2_O and buffered in 0.24 M sodium phosphate (pH 7.4) containing 1 mM sodium-3-(trimethylsilyl)-2,2,3,3-tetradeuteriopropionate (TSP; Cambridge Isotope Laboratories, Andover, MA, USA) and 0.02 M sodium azide. Samples were analyzed using a DRX Avance II+ spectrometer interfaced to a 5-mm TXI ATMA probe (Bruker BioSpin GmbH, Rheinstetten, Germany) at a proton frequency of 500.13 MHz. A presaturation pulse sequence for water suppression based on a one-dimensional nuclear Overhauser effect spectroscopy pulse sequence was used to saturate the residual water proton signal (relaxation delay = 2 s, t_1 _= 4 μs, mixing time = 50 ms, presaturation applied during the relaxation time and mixing time). We collected 128 transients into 64k data points over a spectral width of 8,000 Hz at 300K. NMR spectra were processed in ACD 1D NMR Manager (version 8; Advanced Chemistry Development Inc., Toronto, Canada), multiplied by an exponential weighting function of 1 Hz, Fourier transformed, phased, baseline corrected and referenced to TSP at 0.0 ppm. The NMR spectra were integrated using 0.04-ppm integral regions between 0.2 and 9.56 ppm (excluding water resonance between 4.20 and 5.08 ppm). Spectra were normalized to total integrated area to account for differences in concentration between samples and assigned by comparison with previous literature.

### Gas chromatography-mass spectrometry analysis

Dried aqueous phase samples were derivatized by adding 30 μl of methoxyamine hydrochloride solution (20 mg/ml in pyridine; Sigma-Aldrich Ltd, Dorset, UK), vortex mixed for 1 minute then incubated at 25°C for 17 h. Samples were silylated with 30 μL of *N*-methyl-*N*-trimethylsilyltrifluoroacetamide (Macherey-Nagel, Duren, Germany) for 1 h at 25°C [21]. The samples were then diluted by addition of 200 μL of analytical grade hexane prior to GC-MS analysis.

Acid-catalyzed esterification was used to derivatize the organic phase samples. Chloroform-methanol (1:1, 0.25 ml) and BF_3_-methanol (10%; 0.125 ml) was added to the organic phase and incubated at 90°C for 90 minutes. Water (0.15 ml; mQ) and hexane (0.3 ml) were added and the samples vortex mixed for 1 minute and left to form a bilayer. The aqueous phase was discarded and the organic layer evaporated to dryness prior to reconstitution in analytical grade hexane (200 μl) before GC-MS analysis.

All GC-MS analyses were made using a Trace GC Ultra coupled to a DSQ single-quadrupole mass spectrometer (ThermoScientific, Hemel Hempstead, UK). Derivatized aqueous samples were injected splitless onto a 30 m × 0.25 mm 5% phenylpolysilphenylene-siloxane column with a 0.25 μm ZB-5 ms stationary phase (Phenomenex, Macclesfield, Cheshire, UK). The injector temperature was 230°C and helium carrier gas was used at a flow rate of 1.2 ml/minute. The initial column temperature of 70°C was increased by 5°C/minute to 230°C and then increased at a rate of 20°C/minute to 310°C (transfer line temperature = 250°C; ion source = 250°C; electron ionization = 70 eV). The detector was turned on after 240 s and full-scan spectra were collected using three scans/s over a range of 50 to 650 m/z.

The derivatized organic samples were injected with a split ratio of 8 onto a 30 m × 0.25 mm 70% cyanopropyl polysilphenylene-siloxane 0.25 μm TR-FAME stationary phase column (ThermoScientific). The injector temperature was set to 230°C and helium carrier gas was at a flow rate of 1.2 ml/minute. The column temperature was 60°C for 2 minutes, increased by 15°C/minute to 150°C and then increased at a rate of 4°C/minute to 230°C (transfer line = 240°C; ion source = 250°C; electron ionization = 70 eV). The detector was set as above for the ZB-5 ms column.

GC-MS chromatograms were processed using Xcaliber (version 2.0; ThermoScientific). Each individual peak was integrated and then normalized. Overlapping peaks were separated using traces of single ions. Peak assignment was based on mass fragmentation patterns matched to the National Institute of Standards and Technology library and to previously reported literature. Identification of metabolites from organic phase GC-MS analysis was supported by comparison with a FAME standard mix (Supelco 37 Component FAME Mix; Sigma Aldrich).

### Ultra performance liquid chromatography-mass spectrometry analysis

Chromatography was performed using an ACQUITY UPLC System (Waters Corporation, Elstree, Hertfordshire, UK) equipped with an Acquity UPLC 1.7 μm bridged ethyl hybrid C8 column (2.1 × 100 mm; Waters Corporation) that was kept at 65°C and coupled to a Micromass QTof-*Micro*™ with a Z-spray™ electrospray source. The electrospray source was operated in positive ion mode with the source temperature set at 100°C and a cone gas flow of 50 L/h. The desolvation gas temperature was 300°C and the nebuliser gas flow rate was set at 600 L/h. The capillary voltage was 3 kV and the cone voltage was 40 V. The binary solvent system used was solvent A (HPLC grade water, 1% 1 M ammonium acetate (NH_4_Ac), 0.1% formic acid) and solvent B (analytical grade acetonitrile/isopropanol 5:2, 1% 1 M NH_4_Ac, 0.1% formic acid) [22]. The temperature of the sample organizer was set at 4°C. Mass spectrometric data were collected in full scan mode from 100 to 1,350 m/z from 0 to 14 minutes with a scan duration of 0.5 s and an interscan delay of 0.1 s.

The organic phase of liver and serum were reconstituted in methanol-chloroform (2:1, 500 μl). This was further diluted 7.5-fold and 4-fold, respectively, for the different tissues prior to injection onto the C8 column due to variation between lipid concentrations in the tissues (5 μl and 10 μl, respectively). For both the liver and serum samples the column mobile phase was held at 70% solvent B for 0.5 minutes followed by an increase from 70 to 100% solvent B over 0.5 to 6.5 minutes. The mobile phase was then held at 100% solvent B for 3.5 minutes. Between 10 and 10.25 minutes the mobile phase was returned to 70% solvent B held for 3.75 minutes to re-equilibrate the column. The total ultra performance liquid chromatography (UPLC) cycle was 14 minutes. The eluent flow rate was 600 μl/minute.

Tandem mass spectrometry (MS/MS) was used for the identification of selected lipids. MS/MS runs were performed using ESI+ mode and collision energies of 16, 18, 20, 25, 28 V and a mass range of 80 to 1,100 m/z. Other conditions were as described above.

Data were processed using Micromass Markerlynx Applications Manager (Waters Corporation). Each peak was detected, noise-reduced and integrated. The ion-intensities for each peak were detected and normalized. Lipids were identified using the tandem mass spectrometry data.

### Multivariate analysis

Multivariate data analysis was performed using SIMCA-P^+ ^11.0 (Umetrics AB, Umeå, Sweden). NMR data sets were mean-centered and Pareto-scaled prior to analysis. Pareto scaling involves weighting each of the variables by the square root of that variable's variance, minimizing the impact of noise and increasing the importance of low-concentration metabolites in the subsequent analysis. GC-MS data sets were unit variance scaled. Unit variance scaling weights each of the variables by the variable's group standard deviation and therefore does not bias models towards large concentration metabolites. Data sets were analyzed using principal components analysis (PCA) and partial least squares-discriminate analysis (PLS-DA). Metabolite changes responsible for clustering or regression trends within the pattern recognition models were identified by interrogating the corresponding loadings plot. Metabolites identified in the Variable Importance Parameter (VIP)/coefficients plots were deemed to have changed globally if they contributed to separation in the models with a confidence limit of 95% or greater.

## Results

### Clinical chemistry

**Figure 1:**
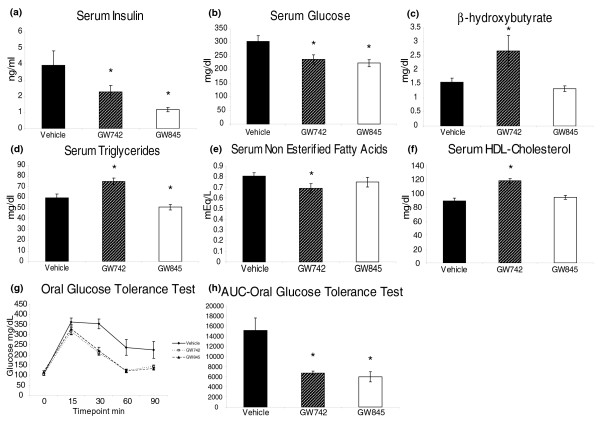
Clinical chemistry measurements from the serum of control, GW610742 PPARδ agonist and GW347845 PPARγ agonist treated *ob*/*ob *mice. **(a) **Serum insulin. **(b) **Glucose. **(c) **β-hydroxybutyrate. **(d) **Triglyceride. **(e) **Non-esterified fatty acids. **(f) **HDL cholesterol. **(g) **Oral glucose tolerance test. **(h) **Area under curve (AUC) oral glucose tolerance test. *P < 0.05 with respect to vehicle control treated animals. Error bars show standard error deviations from the mean.

The concentrations of both insulin and glucose were found to be significantly decreased in both PPARδ and PPARγ agonist treated serum, indicating that the insulin resistant status of the *ob*/*ob *mice is improved by PPARδ and γ activation. This was also confirmed by the oral glucose tolerance test. The concentrations of β-hydroxybutyrate, total cholesterol and HDL cholesterol were increased in the serum of PPARδ agonist treated mice, while non-esterified fatty acids were decreased in concentration. While, serum triglyceride concentrations were increased in PPARδ agonist treated mice, this class of compounds decreased in PPARγ agonist treated mice (Figure 1).

### Metabolomics

^1^H-NMR spectroscopy and GC-MS analysis, combined with multivariate pattern recognition, were used to profile metabolism within the liver, serum and skeletal muscle of *ob*/*ob *mice treated with a PPARδ agonist and a PPARγ agonist. The different analytical techniques had varying sensitivities. High resolution ^1^H-NMR spectroscopy detected 20 to 25 metabolites in both liver and skeletal muscle. GC-MS detected 100 to 150 defined peaks from aqueous phase samples and 30 to 40 defined peaks from organic phase samples. Matching the mass spectra detected with those held in the National Institute of Standards library identified 40 to 60% metabolites for aqueous extracts and approximately 70% for lipids.

Phospholipid targeted UPLC-MS detected 100 to 150 unique metabolite species in positive mode. Identification of metabolite species was performed using MS/MS. Phosphatidylcholines were identified using the phosphocholine head group ion (184 m/z).

**Figure 2:**
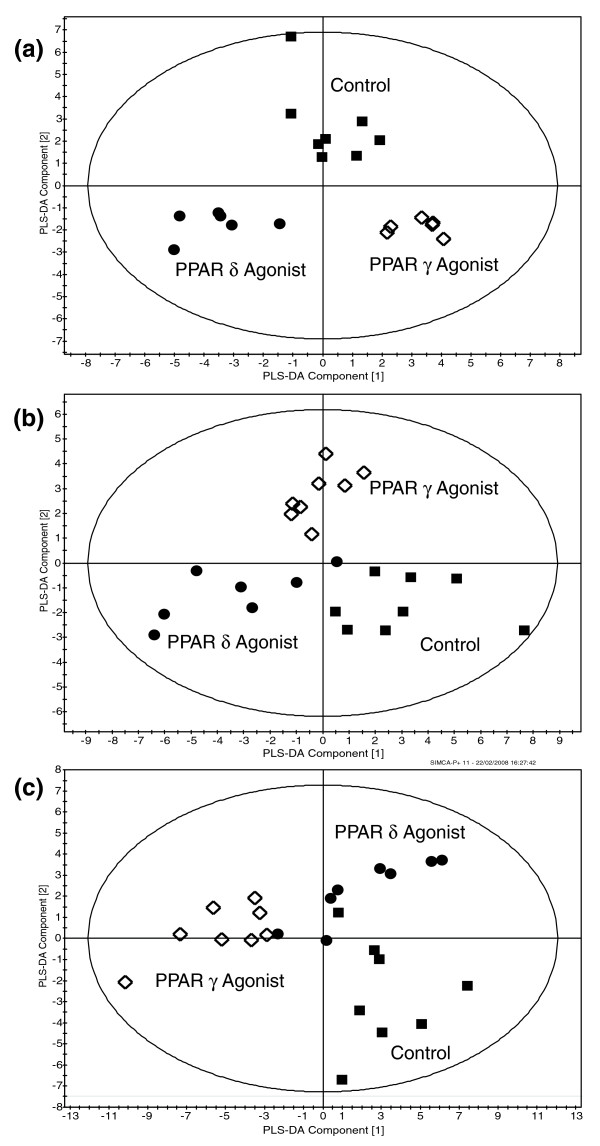
Multivariate analysis of selected GC-MS analysis of key metabolic changes in liver, skeletal muscle and adipose tissue. **(a) **PLS-DA scores plot showing the clustering of GC-MS chromatograms from the organic fraction of the liver from mice treated with either a PPARδ or a PPARγ agonist compared with the control group. Circle, PPARδ agonist treated; diamond, PPARγ agonist treated; square, control (R^2 ^= 0.59, Q^2 ^= 0.82). **(b) **PLS-DA scores plot showing the clustering of GC-MS chromatograms from the aqueous phase of skeletal muscle extracts from mice treated with either a PPARδ agonist or a PPARγ agonist compared with control animals. Circle, PPARδ agonist treated; diamond, PPARγ agonist treated; square, control (R^2 ^= 0.24, Q^2 ^= 0.32). **(c) **PLS-DA scores plot showing clustering of GC-MS chromatograms from the organic fraction of white adipose tissue from PPARδ agonist and PPARγ agonist treated mice following GC-MS analysis. Circle, PPARδ agonist treated; diamond, PPARγ agonist treated; square, control (R^2 ^= 0.58, Q^2 ^= 0.50).

To assess metabolic changes in the dataset, a common processing strategy was adopted throughout the analysis. To investigate metabolite perturbations common to PPARδ and PPARγ activation, PCA and PLS-DA models were built for the individual tissues treating the δ and γ agonists as part of a common group. Although the groups were shown to co-cluster and separate from the control group in this supervised analysis, the majority of the Q^2 ^values (testing model statistical robustness) were low, despite the δ and γ agonist treatment groups separating along the same scores plot axis. While similar changes were detected in the concentration of a large number of metabolites for both treatments, these occurred with different magnitudes (Figure 2). Activation of PPARδ in the liver and skeletal muscle caused a greater magnitude of changes compared to activation of PPARγ. These findings correspond to known tissue distribution of the PPAR subtypes and that the two receptors share a number of common metabolic effects.

**Figure 3:**
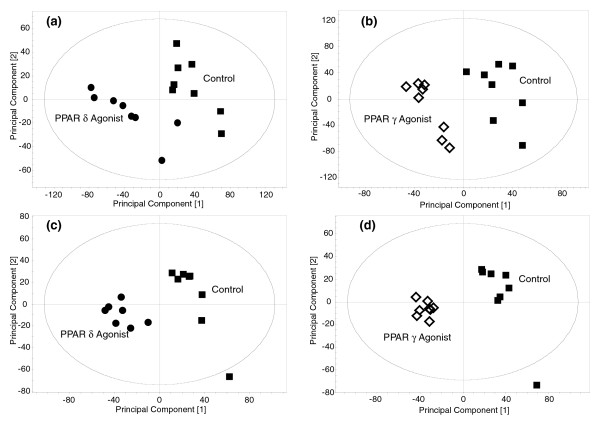
Multivariate analysis of the changes in intact lipid metabolism induced by stimulation of PPARδ and PPARγ. **(a) **PLS-DA scores plot showing the clustering of the UPLC-MS chromatograms from the organic fraction of liver tissue from mice treated with a PPARδ agonist compared with control mice. Circle, PPARδ agonist treated; square, control (R^2 ^= 0.88, Q^2 ^= 0.57) **(b) **PLS-DA scores plot showing the clustering of the UPLC-MS chromatograms from the organic fraction of liver tissue from mice treated with a PPARγ agonist compared with control mice. Diamond, PPARγ agonist treated; square, control (R^2 ^= 1.00, Q^2 ^= 0.85). **(c) **PLS-DA scores plot showing the clustering of the UPLC-MS chromatograms from the organic fraction of serum from mice treated with a PPARδ agonist compared with control mice. Circle, PPARδ agonist treated; square, control (R^2 ^= 0.95, Q^2 ^= 0.82). **(d) **PLS-DA scores plot showing the clustering of the UPLC-MS chromatograms from the organic fraction of serum from mice treated with a PPARγ agonist compared with control mice. Diamond, PPARγ agonist treated; square, control (R^2 ^= 0.99, Q^2 ^= 0.86).

Visual inspection of the ^1^H-NMR spectra and GC-MS chromatograms indicated differences between the control and treated groups. PCA and PLS-DA models were built for the individual tissues comparing the control group with the PPARδ agonist treated group and the control group with the PPARγ agonist treated groups (Figure 3). Metabolites identified in the VIP/coefficients plots as significantly contributing to separation in the models were then considered to have changed globally. Metabolite changes were then compared between agonists (Additional files 1 and 2). The metabolite changes in the individual tissues are considered below.

#### Liver

##### Metabolite changes unique to PPARδ activation

**Figure 4:**
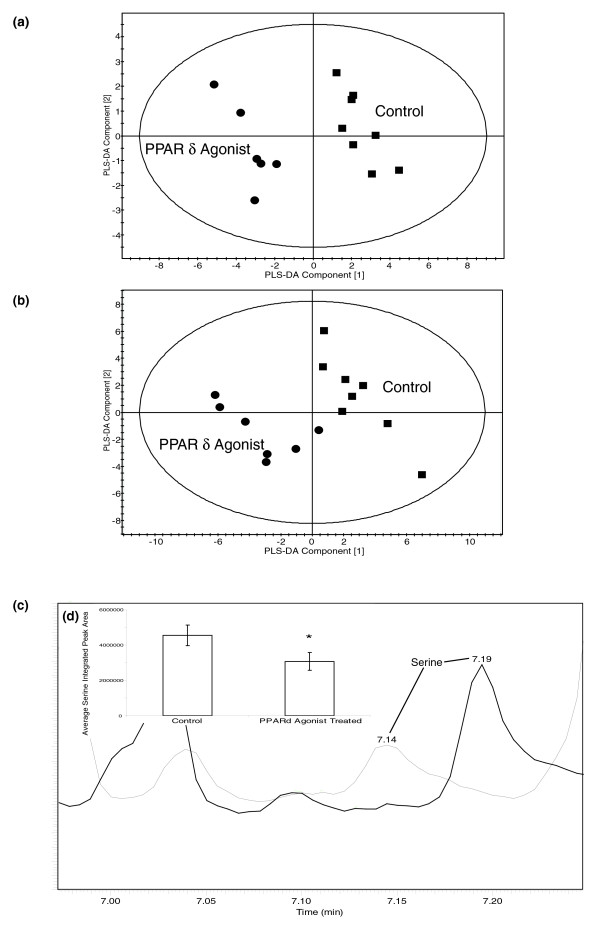
Multivariate analysis of some of the key metabolic changes induced by PPARδ stimulation. **(a) **PLS-DA scores plot showing the clustering of the GC-MS chromatograms from the organic fraction of liver tissue from mice treated with a PPARδ agonist compared with control mice. Circle, PPARδ agonist treated; square, control (R^2 ^= 0.43, Q^2 ^= 0.82). **(b) **PLS-DA scores plot showing the clustering of the GC-MS chromatograms from the aqueous extracts from skeletal muscle from mice treated with a PPARδ agonist compared with control mice. Circle, PPARδ agonist treated; square, control (R^2 ^= 0.69, Q^2 ^= 0.73). **(c) **Comparison of the region of typical GC-MS chromatograms of control skeletal muscle tissue (black) and skeletal muscle tissue from mice treated with a PPARδ agonist (gray) containing the serine peak. **(d) **Bar graph demonstrating the difference in the average integrated area of the serine peak from control and PPARδ agonist treated liver. Error bars show standard error deviations from the mean. *P < 0.05 with respect to vehicle control animals.

While only a decrease in the concentration of the ketogenic amino acid lysine distinguished the animals treated with the PPARδ agonist from the other groups for aqueous soluble metabolites, this group was more readily distinguished by lipid metabolites. The PPARδ agonist produced an increase in the -CH_3 _and -(CH_2_)_n _lipid moieties, detected by ^1^H-NMR spectroscopy. A decrease was detected in the concentrations of 8,11-eicosadienoic acid, cis-10-heptadecanoic acid, myristic acid, myristoleic acid, oleic acid, palmitic acid, pentadecanoic acid and trans-11-eicosenoic acid. The essential fatty acid pathways were also targeted with increases in arachidonic acid, dihomo-γ-linolenic acid, cis-4,7,10,13,16,19-docosahexaenoic acid and a decrease in γ-linolenic acid (Figure 4a).

Given the increased ketogenesis and reduction in triacylglycerides observed in the liver for both agonists, it was deemed important to examine how these metabolic changes were influencing the metabolism systemically by analyzing the blood serum of the animals.

#### Blood Serum

##### Metabolite changes unique to PPARδ activation

Amino acids increased in the PPARδ agonist treated mice serum relative to control were aspartate and isoleucine. The concentration of the ketone body β-hydroxybutyrate was increased, as were the concentration of the tricarboxylic acid (TCA) cycle metabolite fumarate and the carbohydrate catabolite lactate. Fatty acid metabolism was also affected in the serum with increases in the concentration of 2-monostearin, palmitelaidic acid, palmitoleic acid and tetradecanoic acid. The ϖ-6 essential fatty acid pathway intermediate dihomo-γ-linolenic acid was also increased in PPARδ agonist treated mice serum. UPLC-MS analysis highlighted that the concentration of a range of triacylglycerides was found to be elevated in PPARδ treated serum; this was the reverse of the observation upon PPARγ activation, where the concentration of the same triacylglycerides was found to be decreased (Additional file 2).

To examine the fate of the increased serum β-hydroxybutyrate produced by the liver through increased fatty acid oxidation following exposure to the PPARδ agonist, the metabolome of skeletal muscle was examined.

#### Skeletal muscle

##### Metabolite changes unique to PPARδ activation

In contrast to liver tissue amino acid metabolism, glycolysis and the TCA cycle were profoundly affected in the skeletal muscle of PPARδ agonist treated mice. Increases in the concentration of aspartate and α-glycerophosphoric acid and decreases in arginine, glutamine, glycine, methionine, norvaline, serine, glucose, lactate and succinate were detected (Figure 4b, c, d). Fatty acid metabolism was changed in the skeletal muscle of PPARδ agonist treated mice, with an increase in the -CH_3_, COCH_2 _and -(CH_2_)_n _lipid moieties and cis-5,8,11,14,17-eicosapentaenoic acid, elaidic acid and margaric acid. There was a concomitant decrease in palmitic acid. The ϖ-6 essential fatty acid pathway intermediate dihomo-γ-linolenic acid was also increased.

#### White adipose tissue

Given the detected systemic changes in lipid metabolism identified in the *ob*/*ob *mice treated with the PPARδ and PPARγ agonists, and the significant role the adipose tissue has to play in fatty acid metabolism, analysis of fatty acid metabolism in white adipose tissue was conducted using GC-MS of the total fatty acid pool as well as NMR spectroscopy of the aqueous fraction.

For both agonists no change in aqueous metabolism was detected, indicating that the major contributions to changes in aqueously soluble metabolites in serum where associated with metabolic changes in other tissues.

Fatty acid metabolism in white adipose tissue from *ob*/*ob *mice treated with the PPARδ agonist was characterized by a decrease in the concentration of medium carbon chain fatty acids with a concomitant increase in the concentration of the shorter chain fatty acids. For PPARδ agonist treated white adipose tissue an average ratio of the fatty acids C8:0-14:0/C15:0-16:0 = 0.58, whereas for control animals the ratio = 0.53 (*P *< 0.05).

Fatty acid metabolism in white adipose tissue from PPARγ agonist treated *ob*/*ob *mice was distinguished by increased concentration of long carbon chain fatty acids and an increase in the monounsaturated fatty acid products of the Δ-9 desaturase. The ratio of C14:1-16:1 control/C14:1-16:1 PPARγ agonist = 0.87. Analysis by *t*-test demonstrated that the difference between the concentration of fatty acids C14:1-16:1 from control animals and PPARγ agonist treated animals was statistically significant (*P *< 0.005).

## Discussion

A range of complementary metabolic profiling approaches were used to study key tissues involved in type 2 diabetes from *ob*/*ob *mice treated with a PPARδ or a PPARγ agonist to understand the role of PPAR-δ in regulating systemic metabolism. In particular we investigated the core components of the Cori cycle to understand the implications altered liver metabolism has on muscle tissue. While similarities were present between the two agonists, and in particular activation of both PPARδ and PPARγ resulted in an increase in the insulin sensitivity and glucose tolerance of the *ob*/*ob *mice, PPARδ induced a number of unique responses, particularly in liver and skeletal muscle. These findings are consistent with the high level of PPARδ protein expression in these tissues [23]. A decrease was detected in glucose and galactose in all tissues, and fructose in serum and liver from PPARδ agonist treated mice; the decrease in glucose in serum was confirmed by clinical chemistry. Concomitantly, an increase in lactate was detected in the liver and serum of the treated mice, indicating a decrease in hepatic glucose production that has previously been observed following PPARδ activation [18]. It has been suggested that PPARδ activation increases glyceraldehyde-3-phosphate, formed from the 5-carbon sugar phosphates during the pentose phosphate shunt, which can then enter glycolysis [18], explaining the observed reduction of glucose, galactose and fructose in the serum and skeletal muscle.

**Figure 5:**
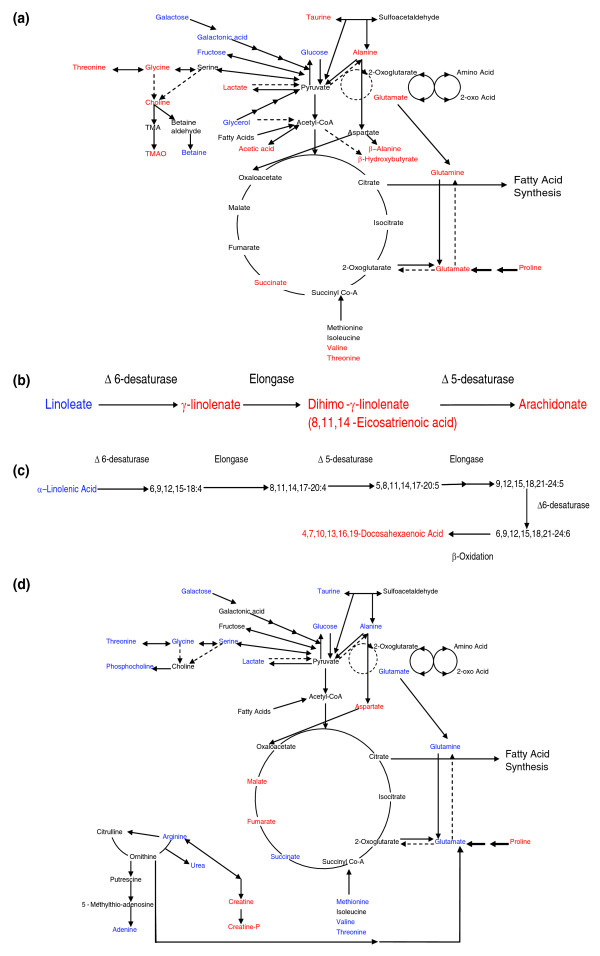
Overview of the key metabolic changes induced by PPARδ stimulation. **(a) **Metabolic pathways altered in PPARδ agonist treated mice liver. Metabolites increased relative to control tissue are in red; metabolites decreased relative to control tissue are in blue. **(b) **Linoleate pathway altered in PPARδ agonist treated mice liver. Metabolites increased relative to control tissue are in red; metabolites decreased relative to control tissue are in blue. **(c) **Linolenate pathway altered in PPARδ agonist treated mice liver. Metabolites increased relative to control tissue are in red; metabolites decreased relative to control tissue are in blue. **(d) **Metabolic pathways altered in PPARδ agonist treated mice skeletal muscle. Metabolites increased relative to control tissue are in red; metabolites decreased relative to control tissue are in blue.

During prolonged β-oxidation of fatty acids in the liver, the production of acetyl-CoA can exceed the capacity of the TCA cycle. The excess acetyl-CoA is converted to β-hydroxybutyrate through ketogenesis in liver mitochondria. The increased liver and blood serum concentrations of β-hydroxybutyrate indicate the PPARδ agonist stimulates ketone body formation for the peripheral tissue, a change that is also observed by clinical chemistry assays. In addition, acetic acid was increased in the treated livers, whilst a decrease in concentrations of non-esterified fatty acids in the serum of PPARδ was indicative of increased tissue oxidative breakdown of fatty acids. The concentration of serum triglycerides was increased in PPARδ agonist treated mice, as they are mobilized for catabolism in liver and muscle. Furthermore, increased fatty acid β-oxidation was apparent in white adipose tissue where there were increased short and medium chain length fatty acids in the PPARδ treated group. These observations are consistent with previous studies showing activation of PPARδ increases fatty acid β-oxidation [24]. Furthermore, activation of PPARγ led to the reverse effect with a decrease in serum triglycerides, consistent with this receptor being involved in regulating white adipose tissue storage of triglycerides and adipocyte expandability [25]. The glucogenic amino acids (those that are precursors of glucose in gluconeogenesis), glycine, glutamate, glutamine, alanine, proline and valine, and the amino acids that are glucogenic and ketogenic, threonine, tyrosine and phenylalanine, were increased in the PPARδ agonist treated livers. In contrast, the concentration of the ketogenic amino acid (those that are broken down to acetyl-CoA and converted to ketone bodies) lysine was decreased (Figure 5a). These changes within the livers of PPARδ agonist treated mice indicate a decrease in gluconeogenesis and an increase in fatty acid oxidation and ketogenesis.

PPARδ agonist treated liver contained decreased linoleate and increased linoleate pathway intermediates, γ-linolenate and dihomo-γ-linolenate, and the pathway end product arachidonate (Figure 5b). The Δ 6-desaturase introduces the initial double bond to linoleate, forming γ-linolenate. The Δ 6-desaturase gene contains a peroxisome proliferator response element and is known to be under PPARα transcriptional control [26]. From this study the desaturase also appears to be under PPARδ transcriptional control; whilst it is worth considering that all pharmacological agonists are likely to exhibit some 'off target' effects, this study has taken into account the high affinity, specificity and extensive characterization of GW610742 for PPARδ, even over the highly related PPARα, which make the compound a very selective tool for the activation of the PPARδ nuclear receptor [27]. Van der Veen *et al*. demonstrated that a dose of 20 mg/kg/day of GW610742 in mice gave an average plasma concentration of 1 μM; given that the specificity of GW610742 for PPARδ is 28 nM compared to 8,900 nM for PPARα and >10,000 nM for PPARγ, then the current study will saturate the PPARδ receptor whilst only minimally activating the other PPAR isotypes [27]. Within the skeletal muscle, linoleate was also decreased and dihomo-γ-linolenate increased but arachidonate was decreased. However, increased arachidonic acid metabolism is not necessarily a contradictory result when PPARδ is activated. The exact balance between the concentrations of these pathways presumably arises from the balance between increased β-oxidation and the actual activity of the synthetic pathway across the different tissues, as well as potential cross-talk between the three different PPARs. Thus, while synthesis of polyunsaturated fatty acids may be increased by PPARδ stimulation, increased β-oxidation will also deplete intermediates, and a new steady state will be achieved.

The α-linolenic acid essential fatty acid pathway was also altered in the liver of PPARδ agonist treated mice (Figure 5c). There was a decrease in the initial metabolite in the pathway, α-linolenic acid and a concurrent increase in the pathways product 4,7,10,13,16,19-docosahexaenoic acid. Two steps in the pathway are again catalyzed by the Δ 6-desaturase. Also, the final step in the pathway, the formation of 4,7,10,13,16,19-docosahexaenoic acid from 6,9,12,15,18,21-24:6, occurs via β-oxidation, which is upregulated in the livers of PPARδ treated mice. Nevertheless, as the intermediates in the pathway were not detected, the exact target of PPARδ cannot be identified unambiguously from these data.

PPARδ mRNA is expressed in skeletal muscle at 10-fold higher levels than PPARα mRNA and 50-fold higher levels than PPARγ mRNA [11]. The receptor is preferentially found in oxidative rather than glycolytic myofibers [11]. A major metabolic change exhibited by the PPARδ treated skeletal muscle was a decrease in the concentration of the majority of the observed amino acids (Figure 5d). Since skeletal muscle lacks glucose-6-phosphatase, the amino acids will not have been used as substrates in gluconeogenesis. An alternative fate for the amino acids is as substrates for the TCA cycle, which was also affected. The increased demand for TCA cycle substrates was also apparent from the decrease in succinate and concomitant increase in fumarate and malate. Succinate is the substrate for complex II of the electron-transport chain, which catalyses the formation of fumarate and reduces coenzyme Q. PPARδ activation increases mitochondrial biogenesis, expression of electron transport chain components, such as cytochrome c, cytochrome c oxidase and complex II, and induces muscle fiber type switching to type I fibers [12]. Within the treated skeletal muscle the concentrations of adenine were decreased and those of adenosines and ribose sugars from the adenosines were increased. Therefore, the decrease in amino acids may relate to increased oxidative metabolism occurring in this tissue. In addition, we detected an increase in the concentration of creatine and phosphocreatine in muscle tissue, which reflects the high energy phosphate buffering capacity of the cell in addition to the increase in ATP also detected. These changes are accompanied by a decrease in lactate concentration and increased β-oxidation, indicating a reduction in glycolysis and a switch to more oxidative metabolism. PPARδ activation has been implicated as a cause of skeletal muscle atrophy [17]. As demand for amino acids increases, one mechanism indicated by our results is that proteins are broken down to supply substrates for the TCA cycle.

PPARδ activation also reduced the degree of saturation of fatty acids in the skeletal muscle of the treated *ob*/*ob *mice. Palmitate and stearate concentrations were found to be decreased and concentrations of their monounsaturated forms, palmitoleate and oleate, were increased. The enzyme catalyzing these reactions, stearoyl-CoA desaturase, is under PPAR expressional control [28].

Activation of PPARδ further improved the dyslipidemic state in the *ob*/*ob *mice by increasing the serum HDL cholesterol concentrations. Activation of PPARδ increases the expression of the cholesterol efflux pump ATP-binding cassette transporter1, promoting the efflux of cholesterol from peripheral tissues, which may lead to the observed increase in HDL cholesterol [9].

The alterations in fatty acid metabolism detected in the white adipose tissue of PPARδ and PPARγ agonist treated mice were markedly different. PPARγ activation resulted in an increase in the concentration of longer chain fatty acids, which was indicative of fatty acid synthesis and elongation. However, PPARδ activation in white adipose tissue decreased the concentration of the longer chain fatty acids and simultaneously increased the concentration of the shorter chain fatty acids, indicative of an increase in fatty acid β-oxidation resulting from PPARδ activation. This suggests a mechanism by which PPARγ activation in white adipose tissue may increase the tissue's ability to sequester fatty acid in a safe repository. PPARδ activation in the same peripheral tissue appears to upregulate β-oxidation and may, therefore, suggesting the mechanism by which activation of the nuclear receptor increases the clearance of circulating free fatty acids is increased β-oxidation as detected in this present study.

## Conclusions

A global summary of the observed changes leads to the conclusion that PPARδ activation generates a systemic change in energy balance in which the Cori cycle is profoundly affected. A decrease in hepatic glucose production produces an increase in hepatic and circulating lactate concentrations and a drop in circulating blood glucose; under these conditions, hepatic metabolism begins to favor fatty acid β-oxidation and ketogenesis, with ketone bodies released into circulation to maintain energy supply to peripheral tissues. Furthermore, glucose is decreased within skeletal muscle alongside increased TCA cycle intermediates but without an observed increase in lactate, correlating with the observed increase in oxidative metabolism. Therefore, the activation of PPARδ produces a marked switch from the Cori cycle to ketone and fatty acid metabolism between the liver and oxidative skeletal muscle, which may contribute to the observed improvement in insulin sensitivity.

In conclusion, to understand the global physiological and pharmacological effects of PPARδ activation, which may give rise to further applications for PPARδ agonists, compound treatment studies have been performed on an *ob*/*ob *mouse background. The combined metabolomic study of liver, skeletal muscle and serum identified multiple changes in metabolism in the PPARδ agonist treated mice. These changes showed that PPARδ activation profoundly affected glycolysis, gluconeogenesis, the TCA cycle and linoleic acid and α-linolenic acid essential fatty acid pathways; many of the changes were found to correlate well with known PPAR controlled gene expression. While some of these metabolic perturbations could be induced by a selective PPARγ agonist, there were also specific changes associated with PPARδ, demonstrating the complexity of the PPAR system and cross-talk between different receptors when considering systemic metabolism.

## Abbreviations

GC: gas chromatography; HDL: high-density lipoprotein; MS: mass spectrometry; MS/MS: tandem mass spectrometry; NMR: nuclear magnetic resonance; PCA: principal components analysis; PLS-DA: partial least squares-discriminate analysis; PPAR: peroxisome proliferator-activated receptor; TCA: tricarboxylic acid; TSP: sodium-3-(trimethylsilyl)-2,2,3,3-tetradeuteriopropionate; UPLC: ultra performance liquid chromatography: VIP: Variable Importance Parameter.

## Competing interests

The authors declare that they have no competing interests.

## Authors' contributions

LDR carried out the metabolomic and chemometric studies, and drafted the manuscript. DGH and DWA coordinated the animal study and sample collection. JNH participated in intellectual discussion of the data. AWN participated in the design of the study, in intellectual discussion and held a supervisory role. JLG participated in the design and coordination of the study, in intellectual discussion, held a supervisory role, cleaned the mass spectrometer and helped to draft the manuscript. All authors read and approved the final manuscript.

## Additional files

The following additional data are available with the online version of this paper: a table of metabolite changes detected by GC-MS and ^1^H-NMR in liver, serum and skeletal muscle of control, and PPARδ agonist and PPARγ agonist treated ob/ob mice (Additional file 1); a table of complex lipid changes detected in liver and serum of control, and PPARδ agonist and PPARγ agonist treated *ob*/*ob *mice using UPLC-MS (Additional file 2).

## Supplementary Material

Additional file 1 Metabolites identified in the VIP/coefficients plots as significantly contributing to separation in the PCA and PLS-DA models built for the individual tissues. The control group was compared with the combined PPARδ agonist and PPARγ agonist treated groups. The R^2 ^and Q^2 ^values for the individual models are shown. Red up arrow indicates a detected increase in metabolite concentration. Down blue arrow indicates a detected decrease in metabolite concentration. Click here for file

Additional file 2 Lipids identified in the VIP/coefficients plots as significantly contributing to separation in the PCA and PLS-DA models built for the UPLC-MS analysis of the organic metabolite fraction. The control group was compared with the PPARδ agonist and PPARγ agonist treated groups from liver and serum. Click here for file
