# Supplementary material for: Increased hepatic oxidative metabolism distinguishes the action of Peroxisome proliferator-activated receptor δ from Peroxisome proliferator-activated receptor γ in the ob/ob mouse
Source: Genome Med. 2009 Dec 7;1(12):115. doi: 10.1186/gm115 (PMC2808731; doi:10.1186/gm115)
Supplement: Additional file 2 — Lipids identified in the VIP/coefficients plots as significantly contributing to separation in the PCA and PLS-DA models built for the UPLC-MS analysis of the organic metabolite fraction. The control group was compared with the PPARδ agonist and PPARγ agonist treated groups from liver and serum. [file gm115-S2.PDF]

Liver

Metabolites increased in PPAR δ agonist treated mice relative to control	Metabolites decreased in PPAR δ agonist treated mice relative to control
PC (32:1)	TAG (16:0/16:1/18:1)
PC (34:2)	TAG (16:1/18:1/18:2)
PC (34:1)	TAG (18:1/18:2/16:0)
PC (36:5)	TAG (18:1/18:0/16:1)
PC (36:4)	TAG (16:0/16:0/21:0)
PC (36:3)	TAG (18:2/18:2/18:3)
PC (36:1)	TAG (16:0/16:0/21:0)
PC (38:6)	TAG (18:2/18:2/18:3)
PC (38:5)	TAG (18:1/20:4/16:0)
PC (38:4)	TAG (18:1/16:0/20:1)
PC (38:3)	TAG (18:1/18:0/18:1)
PC (40:6)	TAG (18:1/16:0/21:0)
TAG (16:1/16:1/16:0)	
TAG (16:1/18:1/14:0)	

Metabolites increased in PPAR γ agonist treated mice relative to control	Metabolites decreased in PPAR γ agonist treated mice relative to control
PC (34:2)	PC (36:2)
PC (34:1)	PC (40:6)
PC (36:4)	TAG (16:1/18:1/18:2)
PC (36:3)	TAG (18:1/18:2/16:0)
PC (36:1)	TAG (16:0/16:0/21:0)
PC (38:5)	TAG (18:2/18:2/18:3)
PC (38:4)	TAG (18:1/20:4 16:0)
TAG (16:1/16:1/16:0)	TAG (18:1/18:1/18:1)
TAG (16:1/18:1/14:0)	TAG (18:1/18:0/21:0)

Serum

Metabolites increased in PPAR δ agonist treated mice relative to control	Metabolites decreased in PPAR δ agonist treated mice relative to control
PC (16:0/18:1)	PC (18:2)
PC (16:0/0:0)	PC (18:0)
PC (18:1/0:0)	PC (34:1)
PC (22:6/18:0)	PC (36:2)
TAG (16:1/18:1/18:2)	TAG (18:1/18:1/18:1)
TAG (16:1/18:1/18:1)	
TAG (18:1/18:2/16:0)	
TAG (18:1/18:1/16:0)	
TAG (16:1/16:0/18:1)	
PC (36:3)	

Metabolites increased in PPAR γ agonist treated mice relative to control	Metabolites decreased in PPAR γ agonist treated mice relative to control
PC (16:0/0:0)	PC (18:0/0:0)
PC (18:1/0:0)	PC (34:1)
PC (34:2)	PC (36:2)
PC (16:0/18:1)	PC (40:6)
PC (36:5)	TAG (16:1/18:1/18:2)
PC (36:4)	TAG (16:1/18:1/18:1)
PC (36:3)	TAG (18:1/18:2/16:0)
PC (36:1)	TAG (18:1/18:1/16:0)
	TAG (18:1/18:1/18:1)
