# Supplementary material for: Increased hepatic oxidative metabolism distinguishes the action of Peroxisome proliferator-activated receptor δ from Peroxisome proliferator-activated receptor γ in the ob/ob mouse
Source: Genome Med. 2009 Dec 7;1(12):115. doi: 10.1186/gm115 (PMC2808731; doi:10.1186/gm115)
Supplement: Additional file 1 — Metabolites identified in the VIP/coefficients plots as significantly contributing to separation in the PCA and PLS-DA models built for the individual tissues. The control group was compared with the combined PPARδ agonist and PPARγ agonist treated groups. The R2 and Q2 values for the individual models are shown. Red up arrow indicates a detected increase in metabolite concentration. Down blue arrow indicates a detected decrease in metabolite concentration. [file gm115-S1.PDF]

PPARγ Serum	PPARγ Skeletal Muscle	PPARγ Liver	PPARδ Serum	PPARδ Skeletal Muscle	PPARδ Liver	
				↑		phosphate
				↓		phosphocholine
				↑		phosphocreatine
	↑			↑		phosphonic acid
		↑	↑	↑	↑	phosphoric acid
↑	↑	↑	↑	↑	↑	proline
	↓					pyrophosphate
↓			↓			ribofuranose
				↑		ribose sugar
	↑			↓		serine
↑	↓	↓	↑	↓	↑	stearic acid
↑		↑	↑	↓	↑	succinate
		↑		↓	↑	taurine
			↑			tetradecanoic acid
		↑			↑	threonate
	↓	↑		↓	↑	threonine
		↑			↓	trans-11-eicosenoic acid
↑			↑			trans-9-octadecanoic acid
		↑			↑	trimethylamine-N-oxide
↑			↑			tryptophan
↑		↑	↑		↑	tyrosine
↑	↑		↑	↓		urea
		↑			↑	uridine
	↓	↑		↓	↑	valine
		0.973			0.978	R2-NMR PCA
		0.782			0.767	Q2-NMR PCA
		0.679		0.424	0.841	R2-NMR PLS-DA
		0.723		0.233	0.723	Q2-NMR PLS-DA
0.281		0.6			0.665	R2-GCMS Aq Metabolites PCA
0.149		0.34			0.138	Q2-GCMS Aq Metabolites PCA
0.345	0.127	0.707	0.489	0.692	0.771	R2-GCMS Aq Metabolites PLS-DA
0.457	0.354	0.911	0.794	0.731	0.915	Q2-GCMS Aq Metabolites PLS-DA
					0.741	R2-GCMS Organic Metabolites PCA
					0.118	Q2-GCMS Organic Metabolites PCA
0.859	0.388	0.584	0.859	0.432	0.432	R2-GCMS Organic Metabolites PLS-DA
0.682	0.413	0.877	0.682	0.479	0.82	Q2-GCMS Organic Metabolites PLS-DA

PPARγ Serum	PPARγ Skeletal Muscle	PPARγ Liver	PPARδ Serum	PPARδ Skeletal Muscle	PPARδ Liver	
↑	↓	↑		↑		fumarate
		↑			↑	galactonic acid
↓	↓	↓	↓	↓	↓	galactose
		↓			↑	γ-linolenic acid
↓		↓	↓	↓	↓	glucose
		↑				glucose-6-phosphate
		↑			↑	glutamate
		↑		↓	↑	glutamine
↑		↑	↑			glyceric acid
		↓			↓	glycerol
		↑	↑	↓	↑	glycine
↑						heptadecanoic acid
↑	↑					isocitrate
		↓	↑			isoleucine
		↑	↑	↓	↑	lactate
↑	↑	↑			↑	lauric acid
↓	↓	↓		↓	↓	linoleic acid
	↑	↓			↓	linolenic acid
		↑			↓	lysine
↑	↑	↑		↑		malate
		↓				maltose
		↓		↑		margaric acid
				↓		methionine
		↓			↓	myo-inositol
	↑			↑		myo-inositol isomer 1
	↓			↓		myo-inositol isomer 2
↑	↑	↑	↑		↓	myristic acid
	↑			↑	↓	myristoleic acid
				↓		norvaline
	↑			↑	↓	oleic acid
		↑				ornithine
			↑			palmitelaidic acid
↑	↑	↑	↑	↓	↓	palmitic acid
	↑	↑	↑	↑	↑	palmitoleic acid
	↑				↓	pentadecanoic acid
		↑			↑	phenylalanine

PPARγ Serum	PPARγ Skeletal Muscle	PPARγ Liver	PPARδ Serum	PPARδ Skeletal Muscle	PPARδ Liver	
				↑	↑	-CH3 moieties
				↑	↑	-(CH2)n moieties
↑			↑			1-monopalmitin
↑						2-monopalmitin
			↑			2-monostearin
		↑			↓	8,11-eicosadienoic acid
↑		↑	↑		↑	acetic acid
	↓			↓		adenine
		↑			↑	adenosine
				↑		adenosines
		↑	↑	↑		α-glycerophosphoric acid
↓			↓			α-hydroxybutyrate
↓	↓	↑		↓	↑	alanine
↓	↓	↑			↑	arachidic acid
↓	↓		↓	↓	↑	arachidonic acid
				↓		arginine
	↓					ascorbate
	↓					asparagine
			↑	↑		aspartate
	↓	↑			↑	β-alanine
		↑	↑		↑	β-hydroxybutyrate
↑			↑			bis-monostearin
↑			↑			butanoic acid
		↑			↑	choline
	↑	↑		↑	↓	cis-10-heptadecanoic acid
	↑	↓		↑	↓	cis-11,14-eicosadienoic acid
↓	↓	↓	↓		↑	cis-4,7,10,13,16,19-docosahexaenoic acid
		↓		↑		cis-5,8,11,14,17-eicosapentaenoic acid
				↑		COCH2
				↑		creatine
	↓					creatine enol
	↓	↑		↓		diethylene glycol
			↑	↑	↑	dihomo-γ-linolenic acid
				↑		elaidic acid
↓			↓			erucate
↓		↓	↓		↓	fructose
